# Cardiac rehabilitation in children and adolescents with long QT syndrome: the RYTHMO’FIT pilot study

**DOI:** 10.1186/s13102-024-00941-2

**Published:** 2024-07-12

**Authors:** Luc Souilla, Sophie Guillaumont, Annie Auer, Gael Metzler, Anne Requirand, Marie Vincenti, Gregoire De La Villeon, Jean-Luc Pasquie, Denis Mottet, Pascal Amedro

**Affiliations:** 1 PhyMedExp, CNRS, INSERM, University of Montpellier, Montpellier, France; 2 CHRU Montpellier, Department of Pediatric and Congenital Cardiology, M3C Regional Reference Centre, Montpellier, France; 3 Pediatric Cardiology and Rehabilitation Unit, Saint-Pierre Institute, Palavas-Les-Flots, France; 4 Department of Physiology, CHU de Montpellier, Montpellier, France; 5 EuroMov Digital Health in Motion, IMT Mines Ales, University of Montpellier, Montpellier, France; 6 Department of Pediatric and Adult Congenital Cardiology, M3C National Reference Centre, Bordeaux University Hospital, Pessac, France; 7 Inserm, U1045, IHU Liryc, Bordeaux Cardio-Thoracic Research Centre, Electrophysiology and Heart Modelling Institute, University of Bordeaux, Pessac, France; 8 Department of Foetal, Pediatric and Adult Congenital Cardiology, M3C National CHD Reference Centre, Bordeaux University Hospital, Haut-Leveque Hospital Avenue de Magellan, 33604 Pessac Cedex, France

**Keywords:** Genetic cardiac conditions, Exercise, Educational early intervention, Patient-centered care, Youth

## Abstract

**Background:**

To assess the feasibility, acceptability, safety, and short-term benefits of a tailored cardiac rehabilitation program for children and adolescents with long QT syndrome (LQTS).

**Methods:**

Eight participants, aged between 6 and 18, with a positive LQTS genotype and impaired cardiorespiratory fitness, were enrolled in a 12-week centre-based cardiac rehabilitation program. The program included supervised exercise training group sessions (aerobic, resistance, and outdoor activities) and patient education workshops. Feasibility, acceptability, and safety of the program were prospectively monitored. Feedback from the parents, children, and professionals involved was collected from qualitative interviews. Short-term effects on cardiorespiratory fitness, muscle fitness, physical activity, and health-related quality of life (HRQoL) were measured between baseline and the end of the program.

**Results:**

Retention (88% with one participant dropping out) and adherence (79%) rates were good, and no cardiac events occurred during the 12-week intervention period. Participants, parents, and healthcare professionals expressed a high level of satisfaction with the program. A significant increase between the beginning and the end of the program was observed for ventilatory anaerobic threshold (21.7±5.2 vs. 28.7±5.1 mL/kg/min, *P*=0.01, effect size=0.89), grip strength, (18±5.3 Kg vs. 20±4.7 Kg, *P*=0.02, effect size=0.90), lower limb explosive strength (142±36.5 cm vs. 148±24 cm, *P*=0.02, effect size=0.90), and parent-reported physical health dimension of HRQoL (65.6±9.75 vs. 84.4±20.35, P=0.03, effect size=0.87).

**Conclusions:**

A 12-week tailored centre-based cardiac rehabilitation program was feasible, acceptable, and safe for children with LQTS. Cardiac rehabilitation for children with LQTS presents a new approach aligned with secondary prevention in youth with cardiac diseases.

**Trial registration:**

The trial was registered at Clinicaltrials.gov (NCT05964322, registration date: 27/07/2023).

**Supplementary Information:**

The online version contains supplementary material available at 10.1186/s13102-024-00941-2.

## Background

Children and adolescents who engage in physical activity experience many health benefits, including reduced cardiometabolic risk [1] and depression, [2] as well as improved cardiorespiratory fitness, better quality of life, [3] and a lower risk of chronic disease [4]. A large proportion of the pediatric population with or without chronic conditions fail to meet the recommended guidelines of 60 min per day of moderate-to-vigorous physical activity [5]. In children with chronic conditions, physical inactivity is associated with lower cardiorespiratory fitness, [6] *i.e.*, an independent marker of all-cause mortality in the adult population [7]. Conversely, children with chronic conditions who have been physically active since childhood are less likely to become sedentary adults, [8] which, can ultimately reduce the risk for all-cause mortality by a third [9].

In children with inherited cardiac disease, promoting physical activity remains challenging because of the risk of sudden death during exercise. This is particularly true for children with long QT syndrome (LQTS), the most common inherited cardiac arrhythmia, which has long been restricted in permissible physical activities. Despite any strong evidence justifying this precautionary approach. Limiting physical activities and sports in youth with LQTS has potentially harmful psychological and physical effects, and may expose them to increased cardiovascular risk in adulthood [10, 11]. The latest sports guidelines for heart disease patients have introduced the notion of shared decision-making, [12] nevertheless, these recommendations have not been routinely transposed to children with LQTS. These young patients remain prone to an early onset of cardiorespiratory and muscle fitness impairment, [13] which, cumulated to the level of anxiety commonly reported in this population, may result in poor quality of life and mental health [14–16].

Cardiac rehabilitation programs have emerged in children with congenital heart disease, reporting excellent safety and efficacy on quality of life, physical fitness, and physical activity [17, 18]. Cardiac rehabilitation is defined by the World Health Organization as “the coordinated sum of activity and interventions required to ensure the best possible physical, mental, and social conditions so that patients may, by their own efforts, preserve or resume their proper place in society and lead an active life”. To the best of our knowledge, cardiac rehabilitation has not been evaluated in youth with LQTS. Yet, modern holistic models of preventive care developed in youth with congenital heart disease [17] could be of interest to children with LQTS, considering their potential need for supervised aerobic and resistance training, therapeutic education, and psychological support [13, 14].

The RYTHMO’FIT pilot study investigated the feasibility, acceptability, safety, and short-term efficacy of a structured cardiac rehabilitation program dedicated to children and adolescents with LQTS.

## Methods

### Study design and population

The prospective RYTHMO’FIT pilot study was performed from May 2022 to March 2023 in a pediatric cardiac rehabilitation centre experienced in clinical trials involving preventive cardiology (Saint-Pierre Institute, Palavas-Les-Flots, France). The study was reported following CONSORT guidelines for pilot studies, providing a checklist [19] [Additional file 1].

Children aged 6 to 18 years with an LQTS, characterized by a QT prolongation in repeated 12-lead electrocardiogram (ECG), and/or an LQTS-causative genetic mutation identified after familial screening, were screened from our recently reported cohort on cardiorespiratory fitness in children with LQTS [13]. Children with impaired cardiorespiratory fitness, assessed by a standardized pediatric cardiopulmonary exercise test (CPET), and defined by a peak oxygen uptake (VO_2 peak_) <80% of predicted values and/or a ventilatory anaerobic threshold (VAT) <55% of predicted values, were eligible for the study [17].

### The RYTHMO’FIT cardiac rehabilitation program

#### Core program

The RYTHMO’FIT program was built using the major components of cardiac rehabilitation, *such* as exercise training, patient education, psychosocial support, and was adapted to the population of children and adolescents with LQTS. The program was an addition to current practice and did not replace any existing treatment. The intervention was described following the template for intervention description and replication checklist (TIDier) [20] [Additional file 2] .

A multidisciplinary group of healthcare professionals specialising in pediatric cardiac rehabilitation was established, including a pediatric cardiologist, an advanced practice nurse, an exercise physiologist, a dietitian, and a psychologist. Based on previous research on congenital heart disease [17] and in the absence of prior safety data on cardiac rehabilitation in the LQTS population, the group drafted a 12-week centre-based program. The content of each component (e.g. exercise training, patient education, and psychosocial support) was discussed using a decision-making approach. All professionals involved in the program were informed of the various stages of program development and invited to give their opinions where appropriate. The final version of the RYTHMO’FIT program included two periods (weeks 1 to 6 and weeks 6 to 12) and three different cohorts of 2 to 3 participants by age group (two for 6-12 years and one for 13-18 years old). The initial visit involved pediatric cardiology consultation, an interview with the exercise physiologist and advanced practice nurse, and the assessment of baseline outcomes (for example, ECG, echocardiography, and CPET). Each session incorporated a consultation with a pediatric cardiologist, completion of a questionnaire on exercise intensity and adaptations, and healthcare visits. The final visit was similar to the initial visit.

#### Exercise training

The 12-week exercise training component of the program was built around achievable goals, adapted exercises, and an emphasis on positive reinforcement. Exercise training sessions were supervised by an exercise physiologist, and took place in an equipped gymnasium and on an outside field. After each exercise session, the exercise physiologist collected patient feedback on any difficulties encountered during the sessions, their levels of satisfaction, and ideas for potential improvement.

Participants’ heart rates were continuously monitored by exercise physiologists using wireless ECG sensors (Auxil ECG; Auxil Science GmbH, Germany) attached to their chests. A pulse oximeter and external defibrillator were available during each exercise session. The pediatric cardiologist was always present in the rehabilitation centre during the exercise sessions. From weeks 1 to 6, two exercise sessions of 60 minutes each per week were held, including one aerobic session and one resistance training session. From weeks 7 to 12, one exercise session of 60 minutes per week was held, which combined aerobic and resistance training. Each session included a 5-minute warm-up, 30 min of individualised aerobic/resistance training, 25 min of group activities (*e.g.* basketball, football, squash), and cooldown and feedback with the exercise physiologist.

Aerobic sessions relied on indoor cycling interval training (2 to 3 repetitions of 10 min bouts with 3 to 5 min of active recovery) at the VAT’s heart rate and workload, determined by baseline CPET assessment. During the program, the workload, duration, and frequency of working bouts progress according to the patient's perceived level of exhaustion. Resistance training sessions were held at the local gymnasium using various materials (e.g. hoops, elastic bands, play mats, and kettlebells) and included two different circuit training workouts (upper and lower limbs) of four exercises each (e.g. push-ups, squats, bear walking). Initially, the children learned the movement with no additional weight. Set and progressive loads were recorded over the weeks and adjusted according to patient characteristics, perceived exhaustion, and movement execution. When the participant was unable to perform the required execution movement properly, personalized support from an exercise physiologist was provided. After completing either aerobic or resistance training, participants engaged in a variety of physical activities adapted. Intensity was monitored using ECG sensors and perceived exhaustion. Activities were diversified and adapted to patients’ spontaneous desires to enhance their engagement and interest. Before each session, the participants voted and decided collectively on the activities proposed by the exercise physiologist. Team games, small competitions, and music were integrated into each session to stimulate enthusiasm and motivation which were evaluated through a short questionnaire and oral feedback at the end of each session. After the last session in week 12, a celebratory snack was organised to end the program on a positive and supportive note. The exercise program is described in detail in Additional file 3 [Additional file 3].

#### Patient education program

During the program construction phase, educational topics relevant to the population of children with LQTS were identified, educational tools (e.g. game-based learning platforms) were selected, and interactive child-friendly presentation slides were designed to promote self-management and problem-solving techniques. An advanced practice nurse in pediatric cardiology holding board certification in patient education supervised the educational program. The educational sessions were delivered to the child and his or her family by a multidisciplinary team (advanced practice nurse, pediatric cardiologist, exercise physiologist, dietician, social worker, and psychologist).

The educational component of the cardiac rehabilitation program was divided into three parts: (1) The first one-hour individual educational session was delivered by the advanced practice nurse during the initial visit (first day of week 1) and aimed to assess participants' needs, objectives, basic knowledge of the disease, and expectations of the education program.

(2) A total of 18 half-hour educational group sessions (2 per week from week 1 to week 6, and one per week from week 7 to week 12) were delivered by the multidisciplinary team, addressing four topics: “my heart”, “my treatment”, “my daily life”, and “support”. Participants were warmly encouraged to participate, engage in discussions, and ask questions related to their lives with the LQTS. A specific group session for parents was set up and led by a pediatric cardiologist and an advanced practice nurse.

(3) The final one-hour individual session at the end of the program (the last day of week 12) was designed to gather feedback from the patient and family and to establish a personalised educational report.

The contents of the different educational workshops are further detailed in the additional file 4 [Additional file 4].

### Outcomes

#### Feasibility, acceptability, and safety

The feasibility of the intervention was determined by (1) recruitment rate (number of participants at baseline vs. number of eligible patients), (2) retention rate (number of participants who completed the 12-week program vs. number of participants who dropped out), (3) adherence rate (number of sessions completed vs. number of sessions offered), and (4) a final self-reported questionnaire which provided patient feedback on the program (scored on a scale of 0 to 5, with comments allowed) and their overall satisfaction (scoring from 0 to 10 by patients and families).

Acceptability was determined from the qualitative data collected during all phases of the program (preparation, initiation, core program, and final assessment). Logistics issues and reasons for not attending the sessions were prospectively recorded. All comments from the participants and healthcare professionals throughout the program were aggregated to refine the assessment of the acceptability of the intervention. At the end of the program, the exercise physiologist and advanced practice nurse provided feedback through brief interviews.

Safety outcomes were prospectively collected from patient enrolment to the final visit (12-week follow-up). Serious and non-serious adverse events were analysed by the data safety and monitoring board members, to determine their relation to the intervention, as previously reported.[17]

The feasibility, acceptability, and safety data were prospectively collected by a single investigator.

#### Secondary outcomes

The evaluation of secondary outcomes included the assessment of cardiac parameters, cardiorespiratory fitness, muscle fitness, physical activity, and patient-reported outcomes at baseline and at the end of the 12-week program.

The cardiac parameters included clinical, ECG, conventional, and 2D-strain echocardiography data. Cardiorespiratory fitness was assessed using a standardised pediatric incremental CPET protocol: 1-minute rest; 3-minute warm-up (10–20 watts) in increments of 10, 15, or 20 watts each minute for 8-12 min; 3-minute active recovery (20 watts); and 2-minute rest [17]. The exercise test was considered maximal when two of the following criteria were reached: respiratory exchange ratio (VCO_2_/VO_2_) ≥ 1, maximum heart rate>85% of maximal age-predicted heart rate, plateau of VO_2_ despite the increasing exercise intensity or the patient's inability to provide a minimum pedalling frequency of 60 revolutions per minute despite verbal encouragement. The following parameters were measured: peak oxygen uptake (VO_2peak_), ventilatory anaerobic threshold (VAT), ventilatory efficiency (VE/VCO_2_ slope), maximum heart rate (HR), oxygen pulse (VO2/HR), and maximal power. The same investigator manually calculated the VO_2peak_ and the VAT using V-slope method. The QT interval was manually measured by a single pediatric cardiologist at peak exercise and every minute of the 5-minute recovery phase, under magnification adjacent to a scale with 20-ms segments by the tangent method [21]. For each measurement of exercise ECG, 3 consecutive QT intervals and their corresponding preceding RR intervals were measured. Raw QT measurements were corrected for heart rate using the Bazett Formula (QTc= QT/ √RR, where QT and RR intervals were measured in seconds).

Muscle fitness was determined by assessing the muscle architecture and strength. Muscle architecture was evaluated using a muscular ultrasound technique, on the patient in dorsal decubitus, as previously described [13]. Four parameters were measured: (1) the anatomical cross-sectional area, (2) the pennation angle of vastus lateralis, (3) the muscle thickness of vastus lateralis, and (4) the fascicle length of vastus lateralis, which was assessed from longitudinal analysis with linear extrapolation of the length of the part of the fascicle that was not visible on the image. Five measurements were performed by a single operator for each parameter. The minimal and maximal values were excluded, and the mean of the three remaining values was calculated. Measures were considered valid when the coefficient of variation was < 5%. ImageJ software was used for image analysis. The maximal isometric strength of the lower limb was assessed using a knee extension test with an Easy Force dynamometer (Meloq AB, Sweden). Easy Force is a belt-stabilised handheld dynamometer attached to a support and then linked to the participant's ankle, which continuously records the tension force. The strap was positioned perpendicular to the anterior or posterior aspect of the tibia 5 cm proximal to the medial malleolus. The participants were seated on the edge of the examination table with the knee bent at 60° to avoid knee pain. Participants performed one submaximal trial, followed by three maximal extensions with a one-minute rest period after each trial. The maximum isometric strength of the upper limb was assessed using a handgrip test [13]. The subjects were seated on a chair without an armrest, elbow flexed at 90°, and squeezed the handgrip with their right hand for four seconds, as hard as they could. The procedure was repeated three times, with one minute’s rest between each trial. The average of the three measurements for isometric strength of both the upper and lower limbs was calculated. In the event of a variation coefficient of >5%, the participants performed an additional maximal trial. Lower body explosive muscular strength was assessed using a long-standing broad jump. The participants stood behind the jump line, feet together, and vigorously pushed forward as far as possible. If they lost balance or touched an object, they were asked to repeat the jump. The distance from the jump line to the heel landing was measured. The longest jump distance between the two trials was selected.

The level of physical activity was evaluated using a triaxial accelerometer (ActiGraph GT3X, Pensacola, FL, USA). Participants were instructed to wear it at the waist for seven days, except during sleep and water-based activities such as swimming or bathing. We chose an e-poch of 15 seconds and Evenson's equations were employed to set the intensity thresholds (counts/min) [22] to classify into four categories: light, moderate, vigorous, moderate-to-vigorous intensity, and sedentary level. A wearing period of 10 hours per day for at least 3 days was necessary for the analysis [23].

Patient-reported outcomes were evaluated using the self- and proxy-reported health-related quality of life (HRQoL) PesdQL instrument. PedsQL version 4.0, which is a generic questionnaire, has four multidimensional scales: physical functioning (eight items), emotional functioning (five items), social functioning (five items), and school functioning (five items). The three summary scores were as follows: total score (23 items), physical health summary score (eight items), and psychosocial health summary score (15 items). Each item uses a 5-point Likert scale ranging from 0 (never) to 4 (always). Items are reverse-scored and linearly transformed to a 0-100 scale, with higher scores indicating better quality of life. The psychometric properties of the PedsQL have been validated in the French Pediatric population [24].

### Statistical analysis

Participants’ characteristics were presented using medians and quartiles for continuous variables and frequencies and proportions for categorical variables. The change in median differences between baseline and the end of the program was calculated using the Wilcoxon signed-rank test. The effect size was estimated using Cohen’s d measure. Statistical significance was set at 0.05, and analyses were performed using R Studio software. Given the objectives and study design of this pilot study, sample size calculation was not feasible.

## Results

### Feasibility, acceptability, and safety

A total of 36 patients aged between 6 and 18 years were screened for eligibility from our pediatric LQTS database. Of the 19 participants meeting the inclusion criteria, 11 declined to participate for various reasons: lack of interest (*n*=2), family death (*n*=1), transport problems (*n*=2), interference with the school schedule (*n*=4), or no answer (*n*=2).

**Fig. 1:**
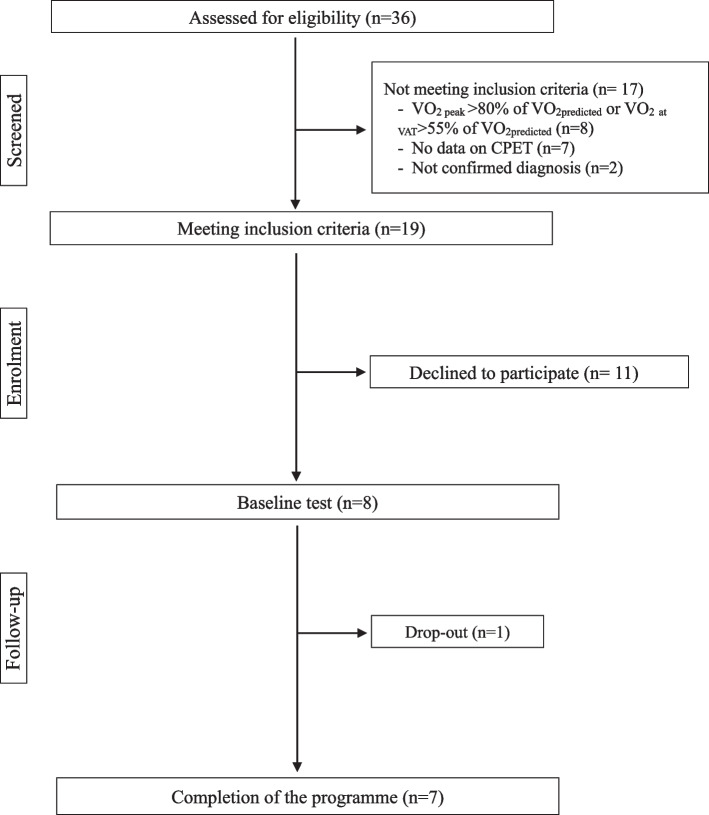
Study flowchart

Finally, eight children (seven boys and one girl) aged between 9 and 17 years (median= 11.5 years old) were enrolled in the study and participated in the baseline assessment during the initiation visit of the cardiac rehabilitation program (Fig 1), corresponding to a recruitment rate of 42%. Participants were carriers of the LQT1 (KCNQ1, *n*=4) or LQT2 (KCNH2, *n*=4) genetic mutations and were all taking beta-blockers (nadolol). The diagnosis of LQTS was made after family screening (*n*=4) or after a cardiac event (*n*=4). The corrected QT interval at baseline ECG ranged from 423 to 536 ms (mean, 474±43.5 ms). Using the Schwartz criteria for LQTS diagnosis [25], four children (50%) had a high probability (≥ 3.5 points), two had an intermediate probability (1.5 to 3 points) and two had a low probability for LQTS (≤ 1 point) (Table 1). One child had a history of refractory ventricular arrhythmias, implantable cardioverter-defibrillator, and cardiac sympathetic denervation (P5, Table 1).

**Table 1:** Population characteristics

**Patient number**	**Age (years)**	**Sex**	**Type of LQTS**	**Genetic mutation**	**Diagnosis**	**QTc interval (ms)**	**Clinical background before rehabilitation**	**Medication**	**LQTS -score diagnosis**^a^
#1	12	Male	LQT1	KCNQ1	Cardiac symptoms	440	Asymptomatic	Nadolol (40 mg daily)	1
#2	9	Male	LQT1	KCNQ1	Cardiac symptoms	497	Asymptomatic	Nadolol (40 mg daily)	5
#3	11	Male	LQT2	KCNH2	Prenatal genetic screening after known causal familial mutation	525	Bradycardia	Nadolol (50 mg daily)	5
#4	17	Male	LQT2	KCNH2	Cardiac symptoms	476	Syncope	Nadolol (80 mg daily)	4.5
#5	9	Female	LQT2	KCNH2	Genetic after known causal familial mutation	536	Refractory ventricular arrhythmia, ICD, cardiac sympathetic denervation	Nadolol (100 mg daily)	8
#6	13	Male	LQT1	KCNQ1	Incidental findings	466	Asymptomatic	Nadolol (70 mg daily)	2
#7	10	Male	LQT1	KCNQ1	Genetic after known causal familial mutation	423	Asymptomatic	Nadolol (40 mg daily)	1.5
#8	15	Male	LQT2	KCNH2	Genetic after known causal familial mutation	424	Syncope, implantable leadless cardiac monitor (REVEAL®)	Nadolol (40 mg daily)	1

*LQTS* Long QT syndrome. *ICD* Implantable cardioverter-defibrillator, *QTc* Corrected QT interval

^a^Schwartz-score diagnostic criteria for long QT syndrome

After the initiation visit and baseline assessment, one adolescent dropped out of the program (participant #8 in Table 1) because of school and transport difficulties, indicating a retention rate of 88%.

Of the 114 sessions organised for the seven remaining participants, 24 sessions were not attended by the children because of interference with the school schedule (*n*=4), transportation issues (*n*=7), absence during holidays (*n*=8), Covid-19 infection (*n*=2) or hospitalisation (=3). Overall, the mean adherence rate was 79%. Two individuals fell within the 50% to 60% adherence range, while five participants had adherence levels above 75%.

Patients’ feedback about the exercise training program found that they were “very satisfied”, in terms of animation and individualized approach of the exercise physiologist, instructions understanding, and socialization during group sessions (“playing, chatting, and fun”). Participants reported being “satisfied” with the exercise session’s intensity and duration. The final questionnaire completed by patients indicated that they were “satisfied” with the intensity and duration of the exercise session, the program's ability to transform their approach to physical activity, and the perceived enhancements on mental and physical well-being. The participants rated the overall exercise training program at 9.75 over 10.

**Fig. 2:**
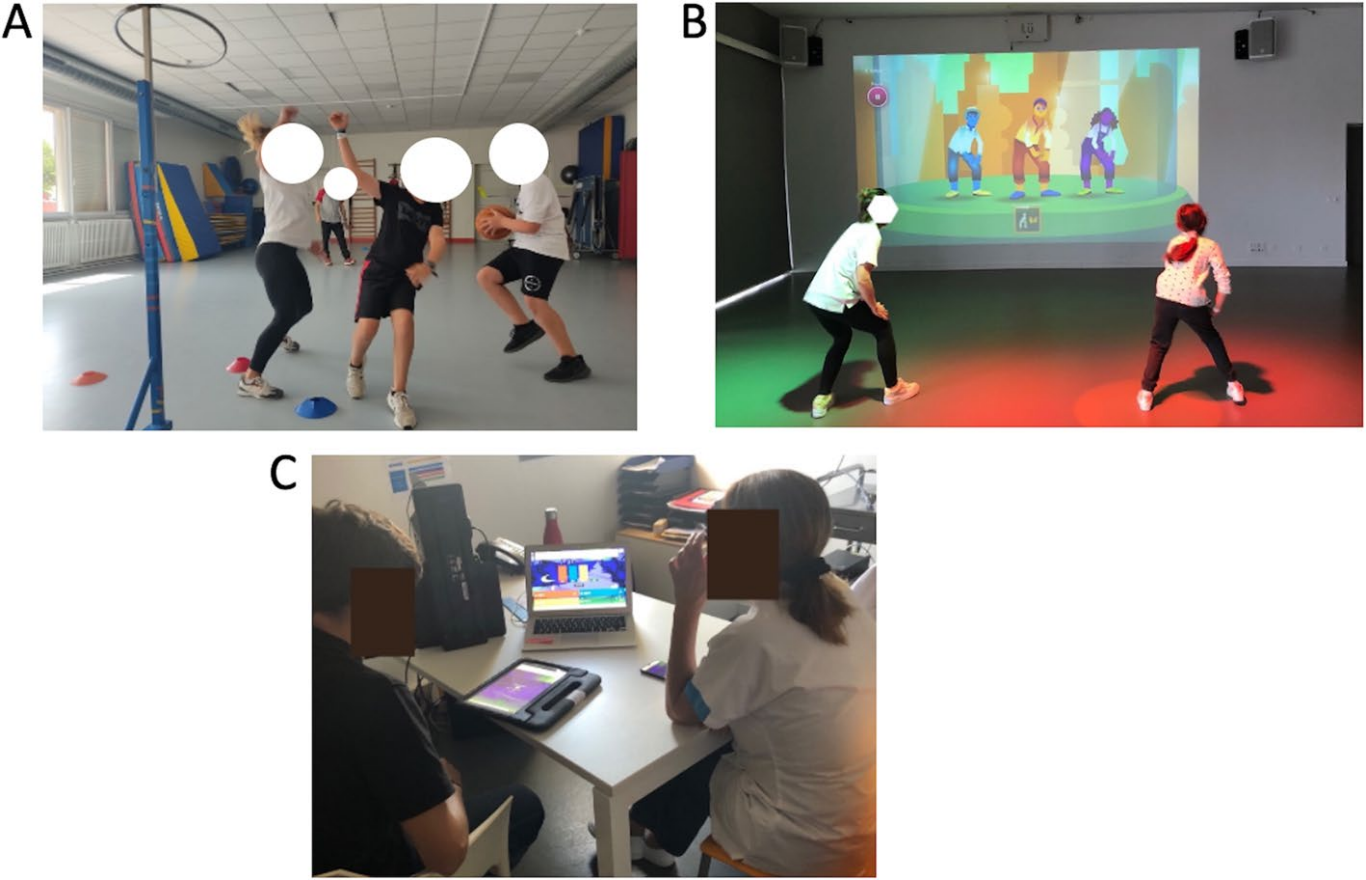
Intervention highlights of the RYTHMO’FIT program. Legend: A- Sports activities group session led by an exercise physiologist, B- Gamification activities led by an exercise physiologist, C- Educational workshops supervised by an advanced practice nurse, the game-based learning platform used was Kahoot (Kahoot! app, https://kahoot.com/). Selected highlights of participants’ feedback: “*This program would have been of great interest during the diagnosis of long QT syndrome where the world is collapsing on us, where football in competition is no longer possible, to support us finding a physical activity project behind.”***,** “*I am truly excited to share this program with children who are dealing with the same disease.”***,** *“This program has not only boosted my son's and my confidence, but it has also granted me the peace of mind to allow him to engage in more activities than before.”*

Patients’ feedback about the education program found that they were “very satisfied”, in terms of improving their understanding of the disease and medical follow-up, as well as managing their daily life. They valued the support provided by the program, the animation and individualised approach of the advanced practice nurse, and educational group sessions. Participants rated the educational program at 9.5 over 10. The most compelling feedback from parents and participants is available in Fig. 2.

In terms of acceptability, the healthcare professionals responsible for implementing the program were very satisfied, given that they had never supervised cardiac rehabilitation in children with LQTS. Twelve sessions were cancelled by the rehabilitation centre because of caregiver vacations or public holidays (deducted in calculating patient adherence). No logistical issues specific to the RYTHMOFIT program were identified compared with the pre-existing cardiac rehabilitation program routinely provided for children with congenital heart disease. In the final interview, the advanced practice nurse underlined the need to refine the educational content for the youngest patients (8-10 years old). The exercise physiologist also advised extending the session to include both aerobic and resistance training, spacing out interventions throughout the day, and implementing a child-friendly heart rate monitoring device, rather than ECG monitoring.

No serious adverse events were reported during the cardiac rehabilitation program. The following non-serious events associated with the exercise sessions were reported: muscle soreness (*n*=4, resistance training) and fatigue (*n*=4, aerobic training). Additional non-cardiac events unrelated to exercise sessions included muscle soreness after physical education sessions (*n*=2), Covid-19 and other infections (*n*=6), hospitalisations due to gastroenteritis (*n*=2), fatigue after prolonged transportation to the hospital (*n*=4), and sleep disorders (*n*=2). During the rehabilitation program, no modification of the routine cardiac follow-up of the patient was observed, and the dosage of beta-blockers remained unchanged.

### Short-term efficacy

The patient characteristics remained unchanged between baseline and the end of the cardiac rehabilitation program (*n*=7) for anthropometric data (weight, height, and body mass index) and functional cardiac parameters at rest (ECG and echocardiography) (Table 2)*.*

**Table 2:** Short-term impact of cardiac rehabilitation

		**Baseline**	**End of program**	***P*-value**	**Effect size**
Clinical outcomes	Weight (kg)	40.7±19.1	41.9±22.5	0.47	0.32
	Height (cm)	150±13.5	150±12	0.10	0.62
	Body Mass Index (kg/m^2^)	19.9±5.2	18.7±5.9	0.81	0.12
	Heart rate (bpm)	59±4.5	62±9.5	0.75	0.19
	Systolic blood pressure (mmHg)	106±6	101±12	0.80	0.12
	Diastolic blood pressure (mmHg)	60±9.5	51±10	0.30	0.41
Echocardiography	Left-ventricle ejection fraction (%)	64.4±7.2	70.5±5.7	0.40	0.28
	IVSd (mm)	5.9±1.6	6.2±0.5	0.83	0.16
	LVEDd (mm)	42.3±4.2	44.6±4.9	0.21	0.48
	Mitral E/A ratio	2.3±0.6	2.2±1.1	0.78	0.03
	Mitral E/e’ ratio	4.8±1.3	4.6±0.8	0.28	0.35
	TAPSE (mm)	23.0±4.0	23.0±4.0	0.58	0.33
	Tricuspid S wave velocity (cm/s)	11±1.5	14±2.5	0.10	0.74
	LV global longitudinal 2D-strain (%)	-21.9±4.7	-23.2±3.3	0.68	0.19
Cardiopulmonary fitness	VO_2peak_ (mL/min)	1554±212.0	1659±295.5	0.20	0.51
	Weight-normalized VO_2peak_ (mL/min/kg)	36.7±13.5	38.9±10.4	0.21	0.49
	Percent-predicted VO_2peak_ (%)	80±25.5	87±15.5	0.27	0.44
	Maximum heart rate (bpm)	146±27	145±14	1	0.03
	Maximum workload (watts)	105±15.0	120±32.5	0.08	0.75
	Maximum O_2_ pulse (mL/beat)	10.4±2.4	11.1±3.2	0.07	0.70
	Maximum systolic blood pressure (mmHg)	110±10.0	120±20.0	**0.03**	0.88
	VAT (mL/min)	1062±291.5	1348±388.0	**0.01**	0.89
	Weight-normalized VAT (mL/min/kg)	21.7±5.2	28.7±5.1	**0.01**	0.89
	Percent-predicted VAT (%)	48±8.6	61±11.5	**0.04**	0.80
	Heart rate at VAT (bpm)	109±9.5	113±17	0.22	0.51
	Workload at VAT (watts)	60±32.5	75±25	0.39	0.42
QTc interval (msec)	QTc at rest	476±58	476±73.5	0.41	0.42
	QTc at 1 min recovery	482±55.5	462±56.0	0.61	0.22
	QTc at 2 min recovery	506±31.0	495±40.5	0.47	0.32
	QTc at 3 min recovery	528±68.0	499±56.5	0.37	0.38
	QTc at 4 min recovery	472±38.0	481±66.0	0.83	0.16
	QTc at 5 min recovery	480±58.0	483±51.0	0.55	0.25
Muscle fitness	Grip strength (kg)	18±5.3	20.±4.7	**0.02**	0.90
	Jump distance (cm)	142±36.5	148±24	**0.02**	0.90
	Peak leg extension (N)	242.3±64.1	242.7±49.3	0.58	0.25
	Mean cross sectional area (cm^2^)	2.5±1.2	2.6±1.6	0.07	0.81
	Pennation angle (°)	12.4±2.4	12.8±1.7	0.68	0.19
	Muscle thickness (mm)	1.4±0.2	1.5±0.3	0.38	0.38
	Fiber length (cm)	6.3±0.5	6.4±1.4	0.37	0.38
Level of physical activity	Light (min/day)	236.8±88.5	222.4±25.2	0.44	0.26
	Moderate (min/day)	33±10.9	26±20.1	0.84	0.32
	Vigorous (min/day)	10.6±2.0	11±3.5	0.84	0.32
	Moderate-to-vigorous (min/day)	43.9±10.1	35.5±23.1	0.84	0.32
	Sedentarity (min/day)	494.1±71.8	478.8±39.7	0.84	0.32
Self-reported HRQoL	Total score	76±22.5	85.8±13.9	0.69	0.13
	Physical	78±18.3	84±12.5	0.42	0.29
	Emotional	80±30	95±30	0.83	0.16
	Relation	90±20	85±12.5	0.36	0.40
	School	85±17.5	85±27.5	0.71	0.30
	Physical health	78±18.3	84±12.5	0.42	0.29
	Psychosocial health	73.3±23.4	87.7±17.5	0.61	0.19
Parent-reported HRQoL	Total score	78.8±21.5	82.4±18.8	0.20	0.51
	Physical	65.6±9.8	84.4±20.4	0.03*	0.87
	Emotional	65±25.0	80±22.5	0.40	0.35
	Relation	70±22.5	95±30	0.13	0.59
	School	75±32.5	75±12.5	0.83	0.09
	Physical health	65.6±9.8	84.4±20.4	0.03*	0.87
	Psychosocial health	81.7±25.8	81.7±18.4	0.45	0.32

*HRQoL* Health-related quality of life, *IVSd* Inter-ventricular septum diastolic diameter, *LVEDd* End-diastolic left-ventricular

diameter, *TAPSE* Tricuspid annular plane systolic excursion, *VAT* Ventilatory anaerobic threshold

Significant *P*-values are marked in bold.

^*^P-values were no longer significant after Holm’s adjustment for multiple comparison on HRQoL variables (*p*=0.18)

In terms of cardiorespiratory fitness, we observed a significant increase between baseline and the end of the cardiac rehabilitation program for the VAT expressed in weight-normalized values (21.7±5.2 mL/min/kg vs. 28.7±5.1 mL/min/kg, *P=0.01, effect size=0.89*) or percent-predicted values (48±8.6% vs. 61±11.5%, *P=0.04, effect size=0.80*). A significant increase in maximal systolic blood pressure during exercise was also observed (110±10.0 mmHg vs. 120±20.0, *P=0.03, effect size=0.88*). Between baseline and final ECG assessment, the QTc interval values at rest, peak exercise, and during recovery remained unchanged.

Muscle fitness significantly improved for upper limb grip strength (18±5.3 Kg vs. 20±4.7 Kg, *P=0.02, effect size=0.90*) and lower limb explosive strength (142±36.5 cm vs. 148±24 cm, *P=0.02, effect size=0.90*).

Actigraph quality criteria were appropriate at baseline (5±1.5 days and 12.51±1.05 hours per day) and at final assessment (5±3.5 days and 12.1±1.32 hours per day). One participant did not fulfil the valid criteria and was excluded from the analysis (*n*=6). No significant change in physical activity level was observed between the baseline and final assessments.

Parent-reported physical health scores of HRQoL significantly improved during the program (65.6±9.8 vs. 84.4±20.4, *P=0.03*, *effect size=*0.87). However, these improvements were no longer significant when taking into account Holm's adjustment for multiple comparisons (*P=0.18*).

## Discussion

The RYTHMO’FIT study reported, for the first time, the implementation of a structured centre-based cardiac rehabilitation program in children with LQTS with good feasibility, acceptability, safety, and short-term efficacy.

This cardiac rehabilitation program dedicated to children with LQTS represents a new care pathway that has demonstrated positive feasibility and acceptability to patients, parents, and healthcare professionals alike. In pediatric LQTS, it is commonly difficult for caregivers and families to strike a balance between the risk of sudden death and the promotion of physical activity. The precautionary principle has long been applied, but restrictions on physical activity in patients with a genetic heart disease may lead to an increased incidence of depression, hopelessness, obesity, and suicidal ideation [26]. The 2020 ESC guidelines have initiated a positive move towards encouraging physical activity in patients with cardiovascular disease, especially by implementing the idea of shared decision-making between patients and physicians [12]. However, these guidelines are intended for the adult population, and it remains difficult to put them into practice during a routine pediatric cardiology consultation. Therefore, cardiac rehabilitation for children with LQTS, through targeted therapeutic education and supervised physical training, presents a new approach to managing these patients, consistent with the development of preventive cardiology in early childhood [27]. Interestingly, the good levels of feasibility and acceptability of the RYTHMO’FIT program are similar to those of rehabilitation programs delivered in clinical routine practice to patients with congenital heart disease [17, 28].

Furthermore, the 12-week exercise program implemented in the cardiac rehabilitation centre was found to be safe, as no adverse cardiac events were recorded, no alterations in the QTc interval were detected, and no adjustments to medical treatment were deemed necessary. This aligns with previous research on appropriately managed children with LQTS [11] and recent evidence supporting the safety of promoting physical activity in this population [11, 29]. A pilot study in asymptomatic adult patients with type 1 LQTS showed no cardiac events and unchanged QTc intervals at rest and during exercise recovery from moderate-intensity exercise programs [30]. Our research suggests that these findings might also apply to children and adolescents with both 1 and 2 LQTS genotypes. Further research on larger cohorts remains necessary to explore individualised responses to exercise based on LQTS genotype and patterns of QT interval adjustments from increased parasympathetic activity during exercise [10, 31]. The absence of cardiac events also aligns with the general principle that the benefits typically outweigh the risks for children with chronic conditions [32]. Overall, these findings may contribute to overcoming common obstacles to physical activity in LQTS, including parental overprotection, lack of motivation, and fear of cardiac events during exercise [33, 34].

In terms of short-term efficacy, this tailored cardiac rehabilitation program improved some major components of cardiorespiratory fitness, muscle fitness, and HRQoL. In terms of cardiorespiratory fitness, the extent of the ventilatory anaerobic threshold (VAT) improvement observed in this study (+24%) aligns with the most effective cardiac rehabilitation programs in children with congenital heart disease (+23%) [18]. Given that VAT is an indicator of physical fitness linked to HRQoL, improving this submaximal parameter through moderate-intensity exercise training stands as an important finding. Children with chronic conditions often experience shortness of breath after moderate exertion, which is a strong indicator of physical deconditioning, and is commonly referred to as impaired VAT. Cardiac rehabilitation programs commonly use the VAT to determine the level of exercise training, as in interval training workouts [17].

Concerning muscle fitness, the intervention also enhanced strength without notable changes to muscle architecture or mass, suggesting neuromuscular adaptations, as seen in healthy children undergoing resistance training [35]. At the end of the program, children with LQTS bridged the gap with healthy children by attaining comparable levels of strength [13, 36, 37]. These positive outcomes are of interest as poor muscle strength is associated with an increased risk of cardiometabolic diseases and impairments in neurodevelopmental and bone disorders [38].

Finally, the RYTHMO’FIT program did not change the self- and proxy-reported HRQOL. The effectiveness of cardiac rehabilitation for children has been previously assessed by measuring HRQoL as the primary outcome [39]. Interestingly, the physical health dimension showed an increase in parents' reports after the program, although not statistically significant after accounting for multiple comparisons, and despite low baseline levels compared to children's self-reports. Classically, in children with cardiac disease, parent-reported HRQoL in the physical dimension is lower than self-reported HRQoL [40] but the correlation between HRQoL scores and cardiorespiratory fitness (VO_2peak_) is better when assessed by parents than by children themselves [41, 42]. These results may also indicate that parents have a more unfavourable view of their child's physical health, which could underline parental overprotection. Parental overprotection could be mitigated at the end of the program to align with self-reported HRQoL.

The levels of moderate-to-vigorous physical activity in children and adolescents with long QT syndrome were not different from previously published data [13, 34] but remained lower than the general population, which typically engages in more than 50 minutes of moderate-to-vigorous physical activity per day [43, 44]. Although some physical activity programs have shown promising results in terms of improving HRQoL and physical activity level [17, 45, 46], others have failed to demonstrate significant positive changes in children with congenital heart disease [47–49].

Hence, certain aspects of the intervention design can be refined to enhance its efficacy for future studies involving larger sample sizes. Transforming the program into a hybrid or home-based model could provide greater flexibility and the opportunity to conduct three sessions per week, which is the most commonly used approach [50]. The recent QUALIREHAB hybrid program designed by our group showed a positive and significant change in physical activity and HRQOL [17]. Incorporating motivational and coaching sessions with an exercise physiologist and emphasizing parental involvement can enhance daily physical activity through autonomous active behaviours [51, 52]. Future studies should examine the long-term maintenance of active behaviours following the program in a larger sample to confirm these results in long QT syndrome.

The RYTHMO'FIT program presents numerous promising opportunities for the future. Exercise training programs could be a way to gradually engage children in physical activities based on individual needs to meet the WHO guidelines [17, 46]. The educational program could play a major role in understanding LQTS mechanisms and treatments. The program may support the shared decision-making process for sports participation, enabling individuals to redefine their pathways after diagnosis and consider other less intense physical activities. Future research should assess the long-term sustainability of physical activity after the program, for instance, by evaluating whether individuals join a sports club or association to maintain active behaviours.

Nevertheless, the RYTHMO’FIT program could be improved to increase its feasibility and acceptability among children and parents. First, only one girl, aged 9, with the highest QTc (536 ms), participated in the program. Previous research has suggested that female sex and the onset of puberty could trigger LQTS-related complications, such as polymorphic ventricular arrhythmia or sudden cardiac death [53]. To confirm the accuracy of our results, future exercise programs should ensure a balanced distribution of male and female participants. Additionally, gender disparities in physical activity seem to persist in the female pediatric cardiac population, affected by lower levels of physical activity, [43] early decline in cardiopulmonary fitness, [54] and, ultimately, poorer HRQoL [40]. Overall, the low recruitment rate and incomplete participation may be due to the centre-based design, which involves frequent transportation and interference with school schedules. To address these issues, transitioning to a hybrid or home-based model may be beneficial as a cost-effective alternative delivery method. Based on the QUALIREHAB hybrid cardiac rehabilitation model designed by our group, future hybrid centre- and home-based programs could pave the way for a new horizon in managing young patients with LQTS [17].

### Study limitations

This pilot study enrolled a small number of patients and was not designed to assess the efficacy of the intervention. No significant increase in peak VO2, *e.g.*, a major predictor of cardiovascular health, has been observed in this study, probably due to the moderate level of exercise training delivered by the program. Indeed, high-intensity physical activity is associated with higher cardiorespiratory fitness [55]. One potential limitation is the lack of QTc measurements during the exercise sessions, given the possible role of regular physical activity in regulating the balance between the parasympathetic and sympathetic nervous systems [56]. Effectiveness also needs to be demonstrated over the longer term. Therefore, future research relying on randomized controlled trials remains necessary to draw conclusions with a higher level of evidence.

## Conclusion

In the RYTHMO’FIT pilot study, the feasibility, acceptability, and safety of a 12-week centre-based cardiac rehabilitation program tailored for children with LQTS were good. The program’s short-term efficacy was also positive in terms of cardiorespiratory fitness and muscle fitness. In line with the emergence of preventive cardiology from an early age, cardiac rehabilitation in children with LQTS opens up a new avenue in the management of these young patients and could be extended to the adult population.

### Supplementary Information

Additional file 1. The CONSORT Checklist for a pilot study, CONSORT Checklist of information to include when reporting a pilot of a feasibility trial.

Additional file 2. The TiDier Checklist, Checklist of the Template for Intervention Description and Replication.

Additional file 3. Description of the 12-week exercise program.

Additional file 4. Content of the educational program of the 12-week exercise program.

## Data Availability

The datasets used and/or analysed during the current study are available from the corresponding author on reasonable request.

## Abbreviations

CPET: Cardiopulmonary exercise test
ECG: Electrocardiogram
HRQoL: Health-related quality of life
LQTS: Long QT syndrome
VAT: Ventilatory anaerobic threshold
VO_2 peak_: Peak oxygen uptake
WHO: World health organisation
