# Supplementary material for: Cardiac rehabilitation in children and adolescents with long QT syndrome: the RYTHMO’FIT pilot study
Source: BMC Sports Sci Med Rehabil. 2024 Jul 12;16:152. doi: 10.1186/s13102-024-00941-2 (PMC11245799; doi:10.1186/s13102-024-00941-2)
Supplement: Supplementary file 3 — Additional file 3. Description of the 12-week exercise program. [file 13102_2024_941_MOESM3_ESM.docx]

**Additional file 3**

Description of the 12-week exercise program

| **Exercise components** | **Duration (D) Sets (St) Recovery (Rc)** | **Specific exercises** | **Intensity (I)**  **Progression (P)**  **Regression (R)** | **Security (S) and intensity monitoring (M)** | **Comments** |
| --- | --- | --- | --- | --- | --- |
| Warm-up | D: 5-10 min | - Joint mobilization - Different movements and slightly increasing pace | Not applicable | Not applicable | - Heart Rate increasing - Participants led the warm-up exercise over time |
| Aerobic interval-training exercises * | D: 35 min  St: 2 to 3 repetitions of 8 to 10 min  Rc: 3 to 5 min active recovery | Cycling on height adjustable ergocycle | I: Heart rate and workload at ventilatory anaerobic threshold  P: Increasing workload and time of working  R: Decreasing workload and adding recovery time | S: Wireless ECG-sensors patches connected to cardiologist tablet  M: Heart rate monitoring by exercise physiologist | - Each session, one participant selects music to listen - Duration and repetition of working bouts increase over the weeks |
| Resistance exercises* | D: 35 min  St: 1 to 2 circuits of four exercises with 45 to 60 s for each exercise, one circuit for upper limb and one for lower limb  Rc: 3 min between circuits, 30 to 45 s between exercises | Exercise for upper limb**:   - Biceps curl - Overhead press - Push-up - Abs crunch - Bench Press - Dips exercise   Exercise for lower limb**:   - Lunge - Squat or wall ball squat - Standing long broad jump - Donkey kick exercise - Side leg raise   Global exercises:   - Bear walking - Spider walking - Plank (3 positions) - Bridge press | I: Perceived exhaustion and feedback from participants. Load weight was recorded over weeks to facilitate progressive load throughout the program  P: Increasing load weight (or elastic band) and working time on each exercise then increasing the number of times each circuit is performed  R: Using free weight exercise, decreasing working time, then changing exercise movement when too hard to acquire | S: wireless ECG-sensors patches connected to cardiologist tablet  M: Participants’ feedback | - Individual challenges, competitions and music were integrated - Personalized support from exercise physiologist for proper execution of movements |
| Adapted activities and sports | D: 25 to 30 min  St: 15 min = motor skills circuit, 15 min = team matches  Rc: Adapted according to duration and intensity of the activity | Adapted activities to desires and needs of the patients:   - Basketball - Football - Volleyball - Hockey - Squash - Badminton - Ping-pong | I: Heart rate at ventilatory anaerobic threshold and perceived exhaustion  P: Adding difficulties in the environment and instructions, minimize recovery time  R: Adding facilities in the environment and instructions, or change the team member, or add recovery time | S: wireless ECG-sensors patches connected to cardiologist tablet  M: Heart rate at rest and participants perceived exhaustion | Exercise physiologist and paediatric cardiologist active participation when necessary   - Competition games and music were integrated |
| Cooldown/ feedback | D: 5 min  St: Not applicable  Rc: Not applicable | Breathing exercise, refuelled and discussions with exercise physiologist | Not applicable | Not applicable | Not applicable |
| Amendments to the program | - We implemented gamification activities through LÜ system (Quebec, LÜ interactive playground, <https://play-lu.com/>), involving 15-minute sessions twice during the 12 weeks. The two sessions did not replace classic activities and sports planned. Only half of participants did gamifications activities due to installation delay and logistics difficulties. - We extended sessions times to 70 minutes from the initially planned 60 minutes in cases where technical issues with the wireless ECG sensors arose | | | | |

*During the last 6 weeks, aerobic and resistance exercises were combined into one session per week.

** Initially, the children learned the movement without any added weight. All movements were adapted according to the participants’ skills, experience, and age.

Abbreviations: D, duration; St, sets; Rc, recovery; I, intensity; P, progression; R, regression; S, security; M, intensity monitoring.
