# Supplementary material for: Cardiac rehabilitation in children and adolescents with long QT syndrome: the RYTHMO’FIT pilot study
Source: BMC Sports Sci Med Rehabil. 2024 Jul 12;16:152. doi: 10.1186/s13102-024-00941-2 (PMC11245799; doi:10.1186/s13102-024-00941-2)
Supplement: Supplementary file 4 — Additional file 4. Content of the educational program of the 12-week exercise program. [file 13102_2024_941_MOESM4_ESM.docx]

**Additional file 4**

Content of the educational program

| **Themes** | **Targeted skills** | **Content and questions addressed** | **Educational tools** | **Healthcare professionals** |
| --- | --- | --- | --- | --- |
| “My heart” | - Understanding cardiac physiology - Understanding LQTS mechanism and trigger events - Understanding the risks associated with LQTS | - How does the heart beat? - Symptoms and diagnosis of LQTS? - Physiopathology of LQTS? - Consequences of LQTS on my body and heart? - How to manage symptoms? - Is this disease serious? | - Workshop and drawings for heart mechanism - Role-playing on “how to detect and manage symptoms?” - Interactive questionnaire on Kahoot platform (<https://kahoot.com/> ) | Advanced practice nurse,  Paediatric Cardiologist |
| “My treatment” | - Understanding beta-blockers and its importance - Knowing where to find the list of medications increasing QT interval - Familiarity with other therapeutic alternatives (e.g., ICD…) | - What is your routine beta-blockers treatment? - Will I stop my treatment during my life? - What are the risks of interrupting treatment? - How to find medication on the list of lqtsdrugs app? - “How to do a CPR?”, “how ICD is implanted and how it works?” | - Real situation, using apps, to find potential harmful drugs/medication - Workshops with children and parents on the importance of taking treatment and medication + Q/A sessions - Role-playing for CPR - Interactive questionnaire on Kahoot platform | Advanced practice nurse,  Paediatric Cardiologist |
| “My Daily Life” | - Understanding sports and physical activity adapted to LQTS and their benefits - Knowing recommended diet and hydration for LQTS - Understanding potential risks associated with pregnancy (adolescents only) | - Can I practice sports and physical activity? - What are the benefits and harms of physical activity? - Which physical activities and sports am I allowed to practice and why? - If I want to practice a non-recommended sport, how to discuss and find a trade-off? - The 10 rules to follow for protecting me during sports practice - How does my lifestyle (healthy eating, sleep) affect my overall well-being? - Which diet may be beneficial or harmful for LQTS? - How does hydration play a key role in LQTS? | - Role-playing on physical activity and desire to practice a non-recommended sports (one cardiologist, one parent, one patient with LQTS) - Meeting with the dietician and the psychologist for hydration and diet advices - Interactive questionnaire on Kahoot platform | Advanced practice nurse,  Exercise physiologist, Dietician, Psychologist |
| “The support” | - Knowing different LQTS association, community care - Being aware of contact information for the paediatric cardiology team. - Preparing the transition for young adults (only for adolescents) - Knowing available resources and online support related to my disease | - How to find further support from patient organizations or websites about LQTS ? - If I have a question related to pregnancy, sports practicing, or smoking, who should I contact? - How to find a adult cardiologist specialized in LQTS when I become an adult? - What are the administrative and medical documents that I should keep with me throughout my life? | - Using a tablet to find websites and patient organizations - Discussion with the specialist nurse about transition and transfer to adult cardiology settings - Workshops with parents for young adult transition and support | Advanced practice nurse, social workers |

Abbreviations: ICD, implantable cardioverter defibrillator; LQTS, long QT syndrome; CPR, cardiopulmonary resuscitation.
